# Diverged landscape of restaurant recovery from the COVID-19 pandemic in the United States

**DOI:** 10.1016/j.isci.2023.106811

**Published:** 2023-05-04

**Authors:** Siqin Wang, Xiao Huang, Bing She, Zhenlong Li

**Affiliations:** 1 School of Earth and Environmental Sciences, University of Queensland, Brisbane, QLD, Australia; 2 Graduate School of Interdisciplinary Information Studies, University of Tokyo, Tokyo, Japan; 3 School of Science, Royal Melbourne Institute of Technology (RMIT), Melbourne, VIC, Australia; 4 Department of Geosciences, University of Arkansas, Fayetteville, AR, USA; 5 Institute for Social Research, University of Michigan, Ann Arbor, MI, USA; 6 Geoinformation and Big Data Research Laboratory, Department of Geography, University of South Carolina, Columbia, SC, USA

**Keywords:** Artificial intelligence, Geographical information science, Geography

## Abstract

The COVID-19 pandemic has imposed catastrophic impacts on the restaurant industry as a crucial socioeconomic sector that contributes to the global economy. However, the understanding of how the restaurant industry was recovered from COVID-19 remains underexplored. This study constructs a spatially explicit evaluation of the effect of COVID-19 on the restaurant industry in the US, drawing on the attributes of +200,000 restaurants from Yelp and +600 million individual-level restaurant visitations provided by SafeGraph from 1st January 2019 to 31st December 2021. We produce quantitative evidence of lost restaurant visitations and revenue amid the pandemic, the changes in the customers’ origins, and the retained visitation law of human mobility—the number of restaurant visitations decreases as the inverse square of their travel distances—though such a distance-decay effect becomes marginal at the later pandemic. Our findings support policy makers to monitor economic relief and design place-based policies for economic recovery.

## Highlights

• It constructs a spatially explicit evaluation of the COVID-19’s effect on restaurants
• More expensive restaurants were most severely affected by COVID-19
• Restaurant markets rebounded the fastest in the Republicans-dominant states
• Restaurant visitations decreases as the inverse square of travel distances

## Graphical abstract

**Figure:**
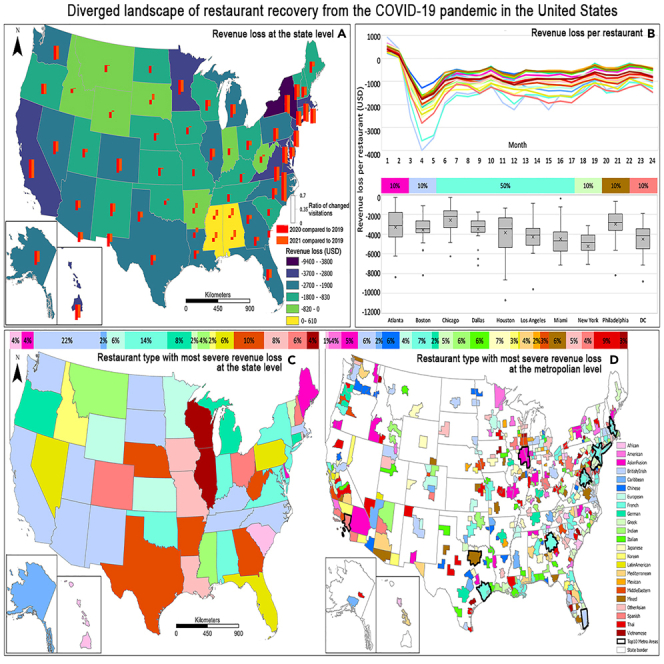

## Introduction

The COVID-19 outbreak in early 2020 has driven the global economy into crisis and imposed catastrophic impacts on various industries. The restaurant industry is one of the most hard-hit sectors during the pandemic due to lockdowns, stay-at-home orders, and other restrictions implemented to control human mobility.^1^ It was estimated that more than 8 million restaurant employees have been laid off or furloughed in the United States (US) since the COVID-19 outbreak, accounting for about two-thirds of workers in all industrial sectors in 2020.^2^ The restaurant industry consists of many kinds of businesses, from nationwide or regional chains to individually owned restaurants. Among these businesses, privately owned restaurants are more vulnerable to the COVID-19 given its relatively small scales and disproportionate composition of low-wage workers.^3^ The US government has given economic relief and financial subsidies (e.g., the Act of Coronavirus Aid, Relief, and Economic Security and the Restaurant Revitalization Fund) to keep private restaurant owners and workers for their livelihoods during the pandemic.^4^ A quantitative evaluation of how the restaurant industry has been recovered from COVID-19 is the primary task for not only providing nuanced evidence for policy making but also tracking the post-pandemic economic revitalization.

However, despite this importance, our quantitative understanding of the effect of COVID-19 on the restaurant industry is surprisingly incomplete. Existing studies mainly drawn from the perspective of hospitality management, rely on qualitative investigation or survey-based analysis to reveal the nexus between COVID-19 restaurant industry and customer behaviors.^3^^,^^4^^,^^5^^,^^6^^,^^7^^,^^8^^,^^9^^,^^10^ What is urgently needed is the quantitative evaluation of the effect of COVID-19 on the restaurant industry at a large spatial and temporal scale, and the comparative analyses across cities and restaurant types. More specifically, first, how does COVID-19 affect the restaurant visitations and revenue gain, and customers’ origins at different spatial levels? And second, whether COVID-19 re-shapes the long-lasting visitation law of human mobility^11^—the number of restaurant visitations decreases as the inverse square of their travel distances—or possibly forms a new normal^12^ of restaurant visitations in the pandemic era? Such an evaluation has far-reaching practical consequences for monitoring economic relief, implementing place-based policies for economic recovery, and preparing for future public emergency.

Here, we address this knowledge deficit and conduct a spatially explicit evaluation of the effect of COVID-19 on the restaurant industry in the US. The design of our study is presented in Figure S1. Drawing on the attributes of +200,000 restaurants from Yelp and +600 million individual-level restaurant visitations derived by SafeGraph^13^ from 1st January 2019 to 31st December 2021, we have three objectives to achieve: 1) to measure the visitation and revenue change of restaurants along the pandemic timeline at the national and state level; 2) to examine the change in the areal characteristics of customers’ origins; and 3) to corroborate if the restaurant visitations after COVID-19 outbreak would follow the long-lasting law of visitations, as stated previously, or form a new normal alongside the multi-wave pandemic dynamics. Our evaluation integrated a geographic information system-based framework with big data mining, web crawling, and cross-referencing techniques, with great generality and reproducibility to be re-employed for evaluating the effect of COVID-19 on other industries and the economic impacts of future public crisis.

## Results

### Changes in the restaurant visitations and revenue

**Figure 1:**
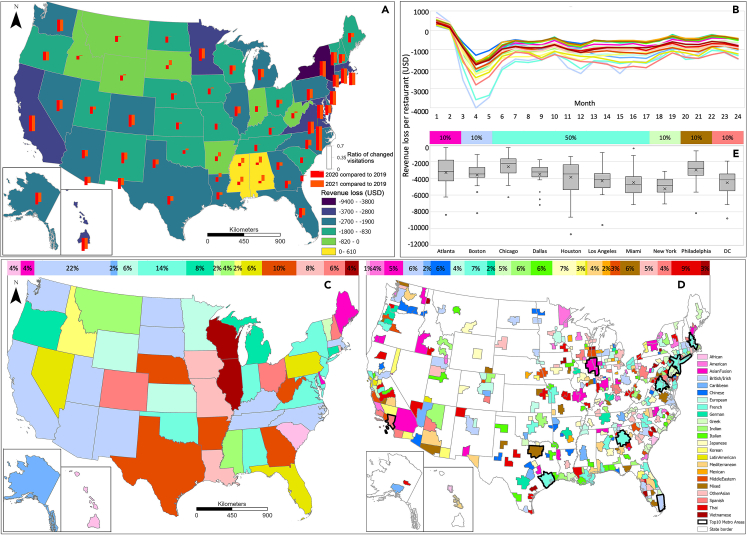
Changes of restaurant visitations and revenue at the national level in 2020-2021 compared to 2019 (A) Changed visitations and revenue of restaurants by state. The height of colored bars indicated the ratio of changed visitations in 2020 compared to 2019 (purple) and in 2021 compared to 2019 (orange). All purple bars (2020/2019) are pointing to the negative direction while a number of orange bars (2021/2019) are in the positive direction; the color of states indicated the changed revenue of 2020 and 2021 compared to 2019 per restaurant. Yellow colored states including Mississippi and Alabama have positive revenue. (B) The temporal change of revenue loss per restaurant for 21 types of restaurants with X axis representing 24 months in 2020–2021. The color of lines represented the type of restaurants, found in the legend of (D). (C) The top restaurant type with the most obvious revenue loss by state (the same legend as [D]). (D) The top restaurant type with the most obvious visitation loss by MSA. The black-outlined MSAs are the top 10 most populated MSAs. The percentage in the horizonal bar on the top of (C) and (D) indicated the proportion of a certain type of restaurants over the total types in all states. (E) The statistical distribution of the revenue loss per restaurant via box plotting in the top 10 MSAs. The percentage in the horizonal bar on the top indicated proportion of a certain type of restaurants over the total types in the top 10 MSAs.

By the end of 2021, all types of restaurants in 48 US states (out of the total 50) experienced revenue loss compared to 2019 (Figure 1A). In 2020, all restaurants had decreased visitations (purple bars in Figure 1A), more obviously in the Democrats-dominant states^14^ along the east coast (e.g., New York and New Jersey) and California. Such states continued to experience visitation loss in 2021, whereas a number of Republicans-dominant states^14^ (e.g., Mississippi, Arkansas, Louisiana, and South Dakota) had restaurant visitations recovered in 2021 over the 2019 baseline. The monthly comparison of restaurant revenue (Figure 1B) between 2020/2021 and 2019 shows that all types of restaurants experienced remarkable revenue loss especially from February to April, 2020; among them, British/Irish and French restaurants had the most significant revenue loss. Then such revenue losses were gradually reduced from May to July, 2020 and remained relatively stable afterward. Among the total 50 states, the largest revenue loss appeared in British/Irish restaurants in 22% of states, followed by French in 14% of states and Middle Eastern restaurants in 10% of states (Figure 1C). Among the total 384 metropolitan statistical areas (MSAs), the largest revenue loss appeared in Thai restaurants in 9% of MSAs, followed by French and Japanese restaurants in 7% of MSAs (Figure 1D). Among the top 10 MSAs with the large population, the average revenue loss is largest in New York (−6,328 USD per restaurant), followed by Miami, Los Angeles, and Washington DC (Figure 1E).

**Figure 2:**
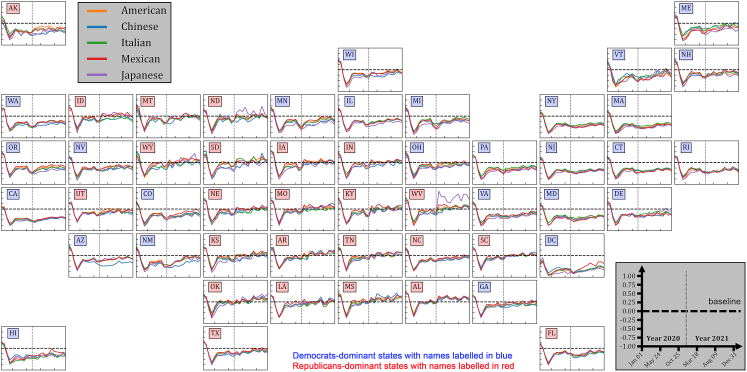
Monthly change of restaurant revenue at the state level in 2020 and 2021 compared to 2019 based on five most popular types of restaurants Within each single graph representing each state, the X axis denotes the pandemic timeline from 1st Jan 2019 to 31st Dec 2021, whereas the Y axis denotes the ratio of monthly revenue change per restaurant in 2020 and 2021 compared to 2019, calculated as the monthly revenue per restaurant in 2020 (2021) minus that in the corresponding month in 2019 then minus 1 (so if the revenue in 2020 is smaller than that in 2019 the ratio will be negative). We selected five most popular restaurants here, represented by colored solid lines, to ensure its visibility within each graph. When the solid line is approaching the dash baseline toward the end of X axis, it means that the restaurant revenue in a certain month in 2020/2021 has been almost fully recovered to the revenue in 2019 (before COVID-19). According to the US 2022 presidential election, Democrats-dominant states have names labeled in blue while Republicans-dominant states have names labeled in red (Levendusky & Pope, 2011)^14^.

We further examined the monthly change of restaurant revenue (Figure 2) and visitations (Figure S2) at the state level based on five most popular restaurant types, coupled with the Kolmogorov–Smirnov (KS)-distance statistics to indicate the (dis)similarity of the restaurant revenue change by type and state (Figures S3 and S4). Some common patterns are observed in Figure 2. In the majority of US states (especially the Republicans-dominant states), the restaurant revenue in the end of 2021 has been almost fully recovered to that in 2019 (solid lines approaching the horizontal dash baseline in Figure 2), or even overcome the revenue in 2019 in some states including Alabama, Mississippi, Arkansas, and Wyoming. Oppositely, in the Democrats-dominant states (e.g., especially California and Washington DC, as well as Maryland, Connecticut, New York, and Rhode Island in the eastern coast), the monthly revenue of these five types of restaurants in the end of 2021 were notably lower than that in 2019, possibly due to the relatively more restricted policy implementation at the state level. The KS-distance statistics provided further quantitative evidence to this finding that the data distributions of monthly revenue in California and Washing DC are significantly different (with KS-distance coefficients above 0.7) to that in other states (Figure S4).

### Changes in the areal characteristics of customers’ origins

**Figure 3:**
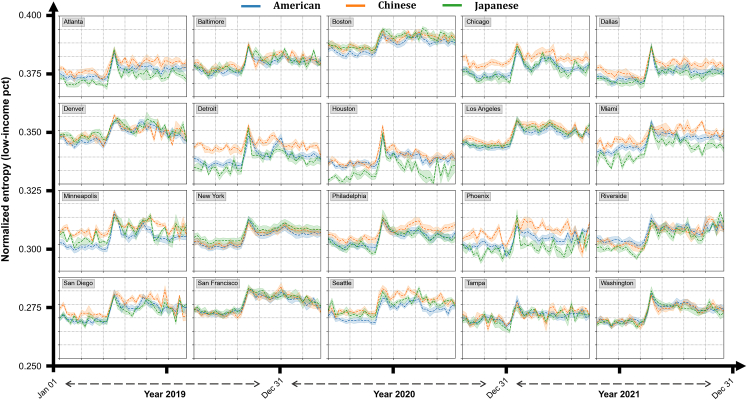
Entropy index used to detect the change in the areal characteristics (the proportion of low-income populations) of customers’ origins after the COVID-19 outbreak Each small graph presents the entropy index of three most popular restaurant types in one MSA; a total of the 20 most populated MSAs are presented here. The enlarged X, Y axis (bold ones) apply to each of the small graph. The X axis denotes the three-year timeline and the Y axis denotes the normalized entropy value calculated based on the proportion of low-income populations over the total. The width buffering around the dash line indicates the 95% confidence intervals.

We used the Entropy Index to reveal the change in the areal characteristics of customer’s origins after the COVID-19 outbreak, in terms of the proportion of low-income populations (Figures 3 and S5) and non-white populations (Figures S6 and S7). In most of the 20 MSAs (except for Houston and Denver), the entropy value (dash lines) in 2021, regardless of the selected popular restaurant types (i.e., American, Chinese, and Japanese), is relatively higher than that in 2019, indicating that the customers’ origins in 2020/2021 become more diverse in income levels than those in 2019. However, the entropy lines presenting the three popular types of restaurants are largely overlapped in Boston, Denver, Los Angeles, New York, San Francisco, Tampa, and Washington DC, reflecting that the origins of restaurant customers living in such cities have no substantial differences in income levels across the three types of restaurants. We also observe a similar pattern for the indicator of non-white populations (Figure S6), indicating that the customers’ origins in 2020/2021 become more diverse in ethnicity. By restaurant type, Chinese restaurants have less diversity in the ethnicity of customers’ origins than American and Japanese restaurants in Los Angeles and Washington DC; while the entropy lines presenting the three popular types of restaurants are largely overlapped in Baltimore, Miami, Riverside, San Francisco, and Tampa, reflecting that the origins of restaurant customers living in such cities have no substantial differences in ethnicity across the three types of restaurants.

### Corroborating the visitation law of human mobility

**Figure 4:**
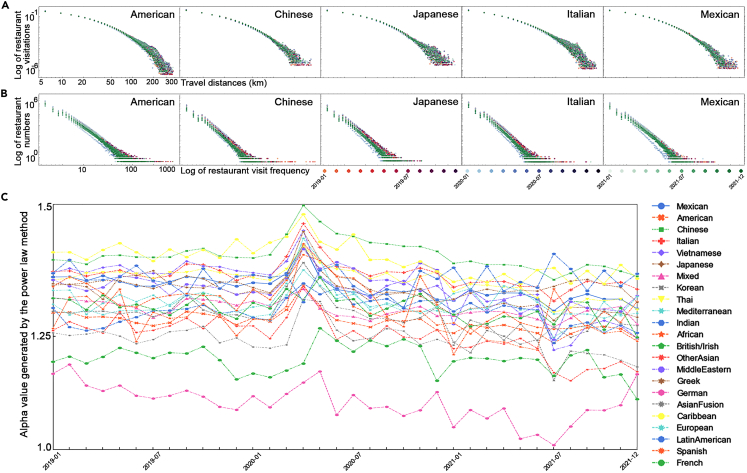
Dynamics among restaurant visitations and travel distances along the pandemic timeline (A) The relationship between travel distances (X) and the log of restaurant visitations (Y). (B) The relationship between the log of restaurant visit frequency (X) and the log of restaurant numbers (Y). The color of dots in (A) and (B) is corresponding to one month in the period from 2019 to 2021, indicated below (B). For (A) and (B), five most popular restaurant types are presented here and graphs for all 23 types of restaurants and 20 MSAs are presented in Figures S8–S11. (C) The alpha value generated by the power law method based on travel distances (km) and the log of restaurant visitations by month and by restaurant type. The alpha value ranges from 1 to 1.5; the larger alpha value means the more significant distance-decay effect, reflecting that restaurants visitors tend to be more sensitive to distances they need to travel to a certain restaurant.

Finally, we examined the relationship between travel distances and restaurant visitations to corroborate if the restaurant visitations after COVID-19 outbreak would follow the long-lasting visitation law of human mobility—the number of restaurant visitations decreases as the inverse square of their travel distances—or form a new normal alongside the multi-wave pandemic dynamics. Figure 4A (Figure S8 presenting all 23 restaurant types) shows the number of restaurant visitations decreased as the inverse square of travel distances, following the assertion of the universal visitation law; however, the magnitude of such a decrease (reflected as the alpha value in Figure 4C) varied along the pandemic timeline and across different restaurant types. For the majority of restaurant types, the alpha value remained stable in 2019, but experienced a sharp increase at the early stage of COVID-19 outbreak and decreased slightly afterward. It indicates that all types of restaurants lost distant customers from February to April 2020 largely due to the COVID-19 restriction policies and people’s self-awareness of viral infections. However, such an effect become marginal in 2021 as the alpha value for most of restaurant types remained similar to that in 2019, although there are some fluctuations for the minority restaurants (e.g., Latin American, African, German, and French), which have not been well recovered. Figure 4B (Figure S9 presenting all 23 restaurant types) provides more evidence to Figure 4A that there are more restaurants with higher visit frequency in 2019 than that in 2020 and 2021 (purple-ramp dots on the right while blue-ramp dots on the left and green-ramp dots in Figure 4B) and the visit frequency was rebounded in 2021 but not fully recovered to the 2019 baseline (Figure S9). This finding is consistent in some MSAs (e.g., Atlanta, Minneapolis, Baltimore, Miami, and Philadelphia); however, other MSAs (e.g., San Francisco, Riverside, San Diego, Phoenix, and Denver) encountered substantial changes of alpha values from July to December 2021 (Figures S10 and S11), reflecting the complexity in the distance-decay effect possibly due to the state-specific COVID-19 policies, as well as the size and urban morphology of metropolitan areas that may influence people’s decision of dining out.

## Discussion

Measuring and mapping the impact of COVID-19 on the restaurant industry, as a crucial socioeconomic sector that contributes immensely to the economy of a nation, are of great importance to policy makers and researchers. Our study contributed a spatially explicit evaluation framework and dataset to assess the recovery of the restaurant industry from COVID-19 in the US. Our findings enrich the existing studies^3^^,^^4^^,^^5^^,^^6^^,^^7^^,^^8^^,^^9^^,^^10^ by distinguishing the restaurant types (e.g., more expensive French and British restaurants), which were most severely affected by COVID-19, delineating the locales (e.g., Republicans-dominant states) where the restaurant markets rebounded the fastest, and revealing the increased diversity of customers’ origins after the COVID-19 outbreak. Furthermore, we find that the visitations of all types of restaurants follow the long-lasting visitation law^11^—the number of restaurant visitations decreases as the inverse square of their travel distances—although all types of restaurants lost distant customers at the early stage of the pandemic; such a distance-decay effect varies across metropolitan areas with state-specific policies and becomes marginal at the later stage of the pandemic.

We introduce some methodological and theoretical advances in the fields of industrial evaluation, spatial economics, and human mobility. By integrating large-scale mobile signal data and points of interests (POI) data together with the techniques of big data mining, web crawling, and cross-referencing algorithms, our spatially explicit evaluation framework can be readily employed to other industries, providing industry-specific and place-based evidence for economic recovery initiatives in response to future public emergencies. For example, SafeGraph data provides POI data in various categories including national parks and liquid stores; future studies could employ our methodological framework to estimate the impact of COVID-19 in tourism, alcohol consumption, and more broad industries for policy making purposes. Theoretically, our comparative perspective on the distance-decay effect before and after the COVID-19 makes it possible to scrutinize how COVID-19 re-shapes the behavior of human mobility, by corroborating the long-standing visitation law longitudinally along the pandemic timeline. Thus, it advances our state-of-the-art understanding of the classic gravity law model^15^^,^^16^^,^^17^ on human mobility that normally concentrates on the spatial dependence of mobility with the temporal perspective and within the context of COVID-19.

Our findings have far-reaching practical consequences for policy implications. Our evaluation results inform policy makers the diverged effect of COVID-19 on the Republicans- and Democrats-dominant states, as well as the locations and types of restaurants that have been most severely affected by COVID-19, which can be used to support the development of state-specific financial programs to subsidize the affected restaurants, areas, and social groups (e.g., low-wage waiters/waitresses who had to work onsite). In addition, our findings are spatially explicit at the fine level of census block groups (CBG), which enhance food delivery from a public health perspective. It provides evidence for neighborhood planning and localization of services—delineating the places as the marketing priorities to meet with the affordability of customers with various socioeconomic backgrounds. Moreover, the changes of mobility behaviors before and after the COVID-19 outbreak vary across urban space and regions, reflecting the human’s perception on place visitations and further linking to the ideation of “revenge travel” that has been observed in the post-COVID cities^18^—as new insights to promote consumption and boost economic recovery locally.

### Limitations of study

Our study limitations are mainly attributed to the data bias existing in the original SafeGraph dataset caused by the concern of data privacy (see STAR Methods). Future efforts can be made to explore other data sources in order to accurately capture the POI visitation patterns of places with low visitation records. In addition, the POI dataset we used only documents the physical visitations of restaurants based on the criterion that a visitation would only be accounted if the duration of that visit to a given POI lasted at least 4 min. It somehow fails to consider restaurant delivery services and take-out services that last shorter than 4 min, which may be supplemented by running a survey or questionnaire to roughly estimate the ratio of dine-in and take-out that can be further used to improve the data and analytical accuracy. It is also worthy to explore the causal effects of other factors beyond the policy implementation on industries (e.g., the change of demographic composition and socioeconomic status of neighborhoods caused by the pandemic) in future studies from different perspectives.

## STAR★Methods

### Key resources table

**Table:** 

REAGENT or RESOURCE	SOURCE	IDENTIFIER
**Deposited data**		

Safegraph data at the aggregated level	https://github.com/giser23/restaurant_analysis	N/A
Yelp data at the aggregated level	https://github.com/giser23/restaurant_analysis	N/A

**Software and algorithms**		

Python and R codes	https://github.com/giser23/restaurant_analysis	N/A

### Resource availability

#### Lead contact

Further information and requests for resources and reagents should be directed to and will be fulfilled by the lead contact, Siqin Wang (s.wang6@uq.edu.au).

#### Materials availability

New datasets generated in this study have been deposited to the project public repository: https://github.com/giser23/restaurant_analysis.

### Method details

#### Restaurant attributes from Yelp

The attributes of restaurants were retrieved from Yelp^19^—a popular customer-review website in North America—via the web crawling technique, including the name, location (latitude and longitude), type and the average cost per customer of 318,890 restaurants in the US. After matching with the POI data retrieved from SafeGraph (detailed in the later section), the total number of restaurants is reduced to 209,612. The attributes of restaurants include ‘UID (unique ID)’, ‘name’, ‘address’, ‘latitude’, ‘longitude’, ‘type’, ‘rate’, ‘cost’, and ‘city’. ‘Rate’ is a score ranging from 1 to 5, reflecting the customers’ review on a given restaurant; a higher rate represents a better reputation. ‘Cost’ is the average consumption of a customer, indicated by dollar signs with four levels ($ to $$$$ representing low to high cost). The quantification of ‘cost’ is detailed in the later section of ‘cost estimation’). ‘Type’ is the original restaurant type classified in Yelp, defined arbitrarily by the mixture of a national cuisine (e.g., Chinese, Korean and Italian), food specialty (e.g., desserts, fried chicken, pizza, and noodle), meal type (e.g., dinner, breakfast, buffet, and cafe), or dietary preference (e.g., vegan, vegetarian, and soul food). We reclassify restaurant types by national cuisine (i.e., by country) because it can be applied to most of restaurants. The detailed procedure of reclassifying restaurant types is elaborated in the later section of ‘restaurant reclassification).

#### Cost estimation

The level of the customer’s average consumption in Yelp is represented by dollar signs ranging from $ to $$$$ (Table S1), indicating an average cost less than 10 USD, 11–30 USD, 31–60 USD and >61. We quantify the level of monetary costs as 10, 20 (the mean of the 11–30 range), 45 (the mean of the 31–60 range), and 60 USD. There are 24% of restaurants (50,388 out of 209,612 that contain not-given costs. We estimate non-given costs based on the weighted average of restaurants with given costs by type and by state (shown as Equation 1) because the socioeconomic disparity of regions affects the cost of restaurants in a certain type. For example, the Chinese restaurants in Manhattan, New York would be more expensive than these in rural New Jersey. After re-estimating the missing costs, restaurant attributes are fully prepared for the later analyses.

(Equation 1) $${ngC}_{s,i}=\frac{\sum_{l=4}C_{i,l}\times n_{i,l}}{N_{s,i}}$$

where ${ngC}_{s,i}$ denotes the non-given cost of a restaurant in a type i in a state of s; $C_{i,l}$ and $n_{i,l}$ denote the estimated given cost and number of restaurants in a type i in a cost level l (e.g., $ to $$$$), respectively; $N_{s,i}$ denotes the total number of restaurants in a type i in a state of s.

#### Restaurant reclassification

The original restaurant type in Yelp was recorded in one column with one to three strings indicating the cuisine of a country, food specialty, meal type and dietary preference; the first string defines the dominant type. We design an algorithm to search these strings in a left-to-right order: if the first string contains the cuisine of a country (e.g., Italian), it will return the country as the dominant restaurant type; if no, it moves to the second and third string to define countries. By doing this, 91% of restaurants (190,746 out of 209,612) are reclassified by country (Table S2). For the remaining 9% of restaurants (18,866 out of 209,612), we set up a hieratical rule to redefine their types: first, if it contains food names that are special in a national cuisine, it will be redefined as that country (e.g., sushi defined as Japanese, taco defined as Mexican, and pizza defined as Italian); second, we manually check the name of a restaurant to reclassify (e.g., a restaurant named as ‘Caribbean Breeze’ is defined as Caribbean); third, all the remaining ones that can be not recognised through the aforementioned procedure (e.g., bakeries, brunch, and diners) are reclassified as mixed. Last, based on the counts of restaurant by country, we keep the major restaurant types as they are (e.g., American, Italian, Mexico, Chinese, Japanese) and regroup the minority by continent (e.g., Afghan and Arabian as Middle Eastern, Argentine and Colombian as Latin American, and Austrian and Belgian as European). The detailed reclassification of restaurant types is presented in Table S2.

#### Restaurant visitation data from SafeGraph

Restaurant visitations were derived from the POI records from SafeGraph^13^—a commercial dataset with the sampling size of 45 million cell phone users in the US—containing the information of total +800,000 restaurants defined in the North American Industry Classification System.^20^ SafeGraph data are retrieved in two different ways, by the location of restaurants (recorded as ‘placekey’ as a unique ID) to generate a point-of-interest (POI) dataset, and by the location of restaurants (as a destination) and census block groups (CBGs, as an origin recorded as ‘CBG ID’) to generate an origin-destination (OD) dataset. The data retrieval is based on the time span of 1 January 2019 to 31 December 2020 by month. This dataset includes the unique identifier (placekey), name, and location of restaurants, the code of census block group where restaurant visitations originated from (GEOID), the number of visitations per week, and the time of visitations. There are total 596,985,934 records of restaurant visitations from 1 January 2019 to 31 December 2021 used in this study. It is worth noting that if the number of visitations per week in a particular restaurant was 1, it had been dropped by SafeGraph due to privacy concern; if less than or equal to 4, it was recorded as 4 though it was likely to be 2, 3 or 4 in reality. To address this data bias, we generated randomized numbers (i.e., integers ranging from 2 to 4) proportionally through a curve estimation algorithm fitting to the number of visitations and frequency (Table S3) via the power form written as below:

(Equation 2) $$Y=aX^{-b}$$

where Y denotes frequency; X denotes the number of visitations; a denotes the coefficient; b is the power.

By scatter plotting the first and second column of Table S3, we generate the below curve estimation (Equation 3) with $R^{2}$ as 0.995 representing a reasonably high model fitness (Figure S14):

(Equation 3) $$Y=3E+08X^{-2.78}$$

Based on this curve estimation (Equation 3), we further estimate the frequency for two, three, and four visitations as 87,040,460 (72.33%), 23,809,892 (19.79%) and 9,491,356 (7.89%) that was used in the randomization function to assign randomized numbers proportionally to the raw records with four visitations (120,341,708).

#### Constructing a spatially explicit restaurant database via cross-referencing

To integrate Yelp’s restaurants with SafeGraph’s POI database, we developed a fuzzy matching algorithm to cross-reference these two datasets, considering their: 1) similarity of restaurant names and 2) spatial proximity. We assume that a Yelp restaurant can be matched to a SafeGraph’s POI if their spatial distances are close enough with identical or similar names. We used the Great Circle distance (GCD) to quantify spatial proximity and the Levenshtein similarity ratio (leR) to quantify name similarity.^21^ The GCD between a restaurant A from Yelp and a POI B from SafeGraph, i.e., GCD(A,B), can be calculated via a haversine formula:

(Equation 4) $$\mathrm{GCD}\left(A,B\right)=2r_{e}\times \sin^{-1}\sqrt{\left(\sin^{2}\left(\frac{\varphi_{B}-\varphi_{A}}{2}\right)+\cos\;\varphi_{A}\;\cos\;\varphi_{B}\;\sin^{2}\left(\frac{\varphi_{B}-\varphi_{A}}{2}\right)\right)}$$

where φ denotes the latitude and φ denotes the longitude. $\left(\varphi_{A},\varphi_{A}\right)$ and $\left(\varphi_{B},\varphi_{B}\right)$ denote the coordinates of restaurants a and b, respectively. $r_{e}$ denotes the radius of the Earth in kilometers (set as 6371 km). The levR between restaurants a and b, i.e., LeR(A,B) with a range from 0 to 1, is calculated based on the Levenshtein Distance (LeD) between A and B, i.e., LeD(A,B):

(Equation 5) $$LeR\left(A,B\right)=\frac{\left({|A}_{n}|+\left|B_{n}\right|\right)-LeD\left(A,B\right)}{{|A}_{n}|+\left|B_{n}\right|}$$

where $A_{n}$ and $B_{n}$ denote the name strings of A and B, respectively. ${|A}_{n}|$ and ${|B}_{n}|$ denote the string lengths of $A_{n}$ and $B_{n}$, respectively. LeD(a,b) is computed via the following formula^21^:

(Equation 6) $$LeD\left(A,B\right)=\left\{ \begin{array}{l} {|A}_{n}|\\ {|B}_{n}|\\ \begin{array}{l} LeD\left(tail\left(A_{n}\right),tail\left(B_{n}\right)\right)\\ \begin{array}{c} 1+\min\left\{ \begin{array}{l} LeD\left(tail\left(A_{n}\right),B_{n}\right)\\ LeD\left(A_{n},tail\left(B_{n}\right)\right)\\ LeD\left(tail\left(A_{n}\right),tail\left(B_{n}\right)\right) \end{array}\right. \end{array} \end{array} \end{array}\right.\;\begin{array}{l} ,if\;{|B}_{n}|=0\\ ,if\;{|A}_{n}|=0\\ ,if\;A\left[0\right]=B\left[0\right]\\ ,otherwise\text{.} \end{array}$$

where x[n] denotes the n-th character of string x (starting with 0) and tail(x) denotes a string of all except for the first character of x. In this study, a match exists between A and B has to satisfy two criteria: 1) GCD(A,B)<0.3km and 2) LeR(A,B)>0.75. We empirically set these two thresholds via trial-and-error experiments. Consequently, 179,250 restaurants (correspondingly 596,985,934 visitation records; Table S4) can be matched up and used as our study population, accounting for 85.5% of the total 209,612 restaurants originally retrieved from Yelp.

#### Thematic mapping

We commenced with generating a series of thematic maps visualising the statistical summary of lost restaurant visitations and revenue in 2020/2021 compared to 2019 by restaurant type, by state and by metropolitan statistical area via cross tabulation, box plotting, and spatiotemporal mapping.

#### Entropy indexing

We then revealed the dynamics of characteristics of customers’ origins before, during, and after the COVID-19 pandemic using the Shannon entropy measurement^22^ for each type of restaurant at different MSAs. Two variables at the CBG-level were investigated: 1) proportion of low-income populations and 2) non-white populations. For restaurant A, we formed a variable set $X=\left\{x_{1},x_{2},...,x_{N}\right\}$ that includes all CBGs of the MSA where restaurant A is located. We then re-ranked X, derived the quintiles, and assigned unique labels given different quintiles for a total of K CBGs (i.e., the origins of visitors) of restaurant A, thus leading to a label set $L_{i}=\left\{L_{i}^{1},L_{i}^{2},...,L_{i}^{K}\right\}$. We described the chaoticness of $L_{i}$ via Shannon entropy^22^:

(Equation 7) $$H\left(L_{i}\right)=-\sum_{j=1}^{T}Ρ\left(L_{i}^{j}\right)\log Ρ\left(L_{i}^{j}\right)$$

where $H\left(L_{i}\right)$ denotes visitation entropy of restaurant A, $Ρ\left(L_{i}^{j}\right)$ denotes the occurrence probability of label $L_{i}^{j}$, and T denotes the number of unique labels in $L_{i}$**.** The entropy $H\left(L_{i}\right)$ quantifies the chaoticness of labels $L_{i}$, thus reflecting how diverse the population restaurant A is able to serve. In light of the scaling issue in Shannon entropy measurement, we normalized $H\left(L_{i}\right)$ by its information length (i.e., number of visitors): $H_{norm}\left(L_{i}\right)=H\left(L_{i}\right)/log\left(N\right)$, where N denotes the total number of visitors to the restaurant A. The higher the value of $H_{norm}\left(L_{i}\right)$, the more diverse population restaurant A serves. We then aggregated the restaurant visitation entropy via restaurant types and MSAs by computing the median values.

#### KS distance statistics

We implemented KS distance statistics, as a nonparametric method that compares two data samples by quantifying the distance between the distributions of two samples,^23^ to investigate how different states present different patterns of restaurant revenue loss and how the revenue loss differs in terms of restaurant types. We first constructed sets of revenue losses, with each set corresponding to a restaurant type. For example, for Chinese restaurants and American restaurants, we respectively constructed ${RevLoss}_{CN}=\left\{R_{1}^{CN},R_{2}^{CN},R_{3}^{CN},\ldots ,R_{k}^{CN}\;\right\}$ with k elements and ${RevLoss}_{US}=\left\{R_{1}^{US},R_{2}^{US},R_{3}^{US},\ldots ,R_{l}^{US}\;\right\}$ with l elements. We investigated whether set ${RevLoss}_{CN}$ and set ${RevLoss}_{US}$ are drawn from the same distribution (two-sample test) by calculating the KS distance between sets ${RevLoss}_{CN}$ and ${RevLoss}_{US}$, i.e., $D_{{RevLoss}_{CN}⟷{RevLoss}_{US}}$, is calculated as:

(Equation 8) $$D_{{RevLoss}_{CN}⟷{RevLoss}_{US}}={\left(\frac{kl}{k+l}\right)}^{1/2}\underset{x}{\sup}\left|F_{{RevLoss}_{CN}}\left(x\right)-F_{{RevLoss}_{US}}\left(x\right)\right|$$

where $F_{{RevLoss}_{CN}}\left(x\right)$ and $F_{{RevLoss}_{US}}\left(x\right)$ denote empirical cumulative density function of set ${RevLoss}_{CN}$ and set ${RevLoss}_{US}$, respectively. In this study, we conducted the KS test for all pairs of restaurant types and states.

#### Gravity law model

Finally, we constructed a gravity law model to examine the relationship between restaurant visitations and travel distances based on two methods—the power law^24^ and exponential algorithms^25^—to reveal the changing distance-decay effect along the pandemic timeline. We first measured the Euclidean distance between the centroid of an origin census block group and a restaurant and classified the numeric measures of distances into bin-distance values (see STAR Methods). Then, we constructed a gravity law model^11^ in two most commonly used algorithms—power law and exponential algorithm:

(Equation 9) $$powerlaw\left(x,k,\alpha \right)=k*x^{-\alpha }$$

(Equation 10) $$exponential\left(x,k,\alpha \right)=k*e^{-\alpha x}$$

where x represents the distance bin; k denotes the smallest distance that people travel to a restaurant; α is a key parameter to reflect the magnitude of distance decay. This model was developed in Python using the *Lmfit* package. The package accepts the initial values for the parameters (k,α) and fit the input data across all distance bins. After the fit, $\alpha_{best}$ would be extracted from the output as the best fit value of the parameter.

## Data Availability

Analyses in this study were conducted in SPSS, ArcGIS Pro, R and Python. Data at the aggregated level and codes used in this study can be accessed from the project public repository: https://github.com/giser23/restaurant_analysis. Data• Data have been deposited at https://github.com/giser23/restaurant_analysis and are publicly available as of the date of publication. Accession numbers are listed in the key resources table. Code• All original code has been deposited at: https://github.com/giser23/restaurant_analysis. • Any additional information required to reanalyze the data reported in this paper is available from the lead contact upon request.
