# Supplementary material for: Diverged landscape of restaurant recovery from the COVID-19 pandemic in the United States
Source: iScience. 2023 May 4;26(6):106811. doi: 10.1016/j.isci.2023.106811 (PMC10156630; doi:10.1016/j.isci.2023.106811)
Supplement: Document S1. Figures S1–S14 and Tables S1–S4 [file mmc1.pdf]

iScience, Volume *26*

Supplemental information

Diverged landscape of restaurant recovery from the COVID-19 pandemic in the United States

Siqin Wang, Xiao Huang, Bing She, and Zhenlong Li

SI Appendix

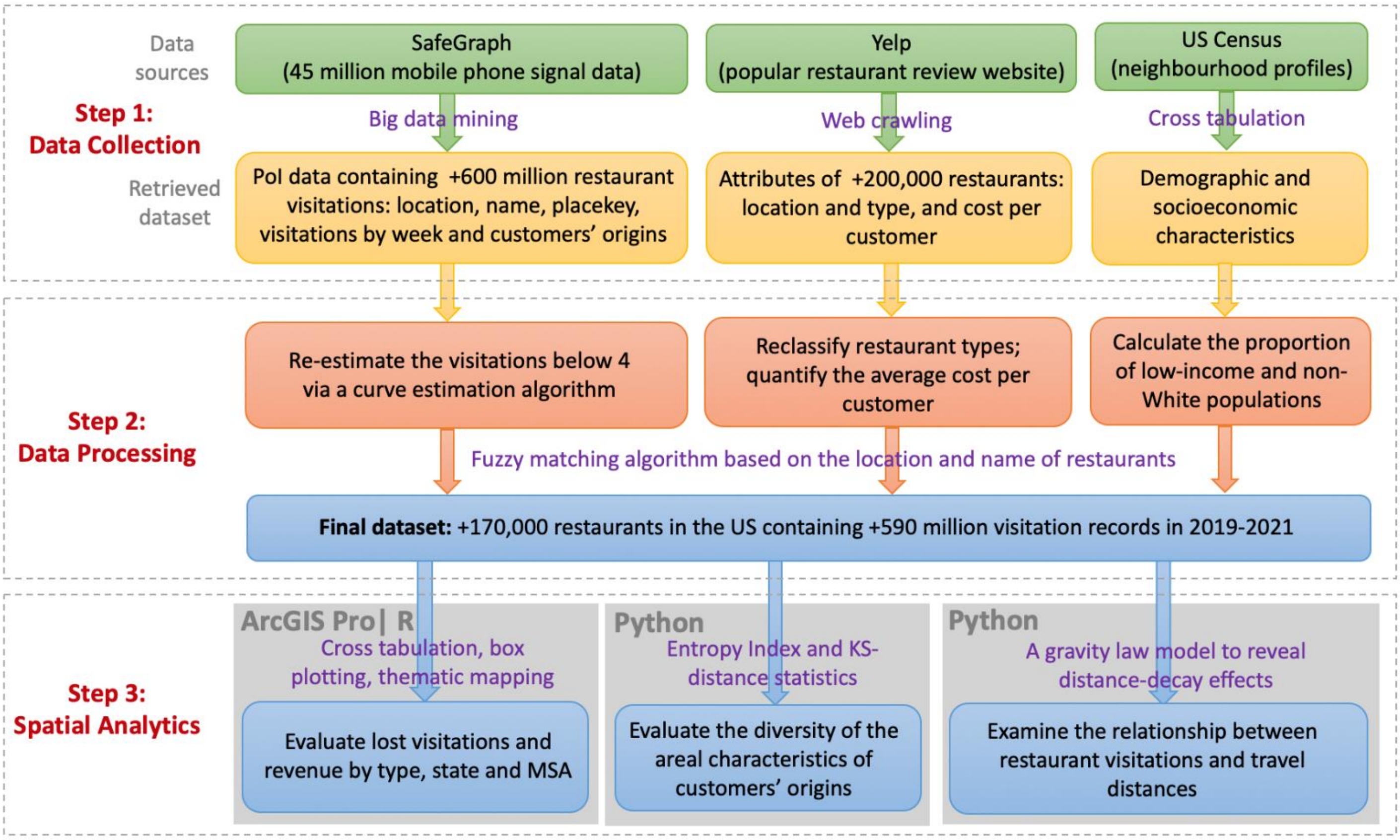

Figure S1. Study design and analytical framework

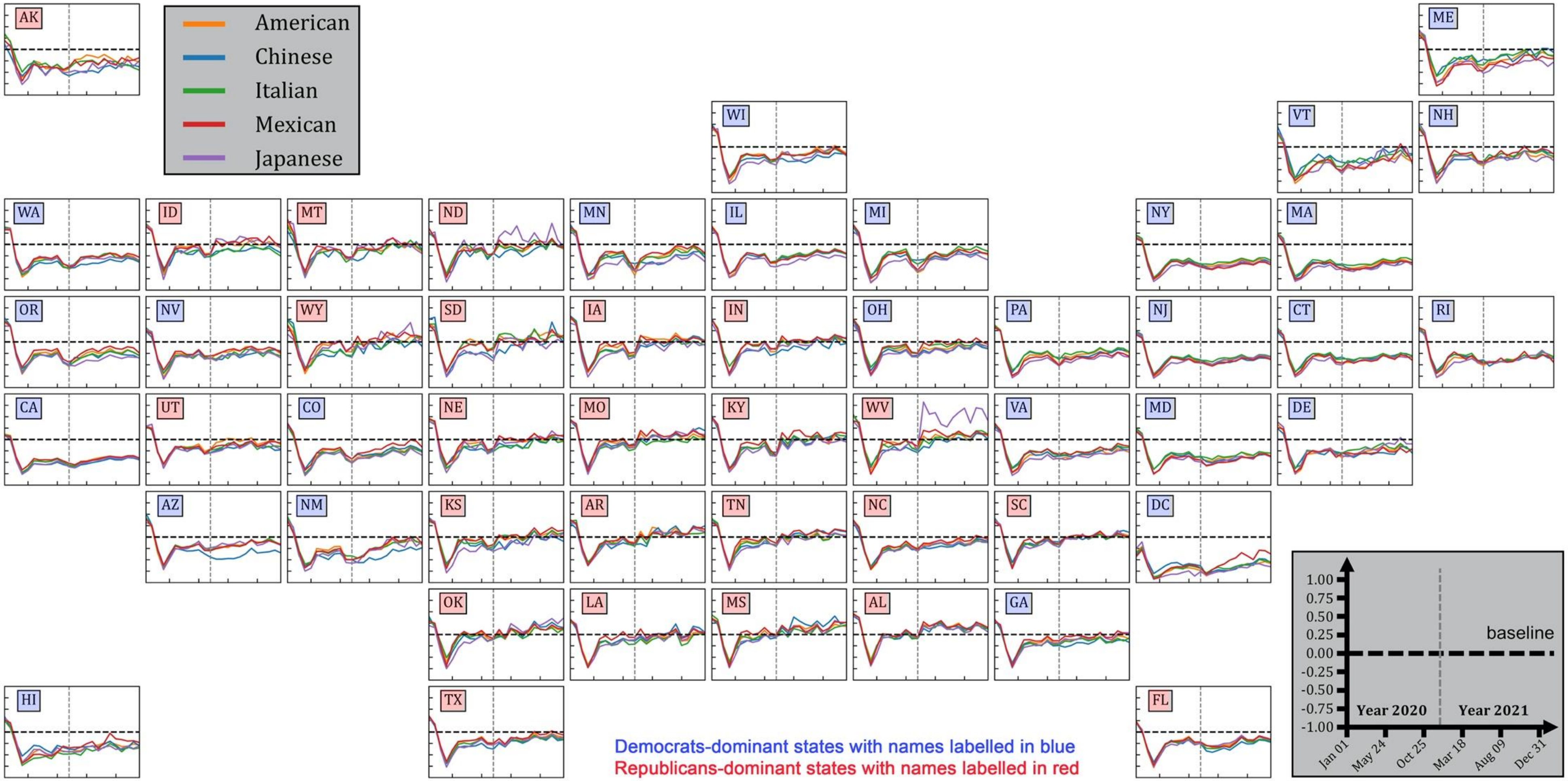

Figure S2. Changes of restaurant monthly visitations at the state level in 2020 and 2021 compared to 2019 based on five most popular restaurants

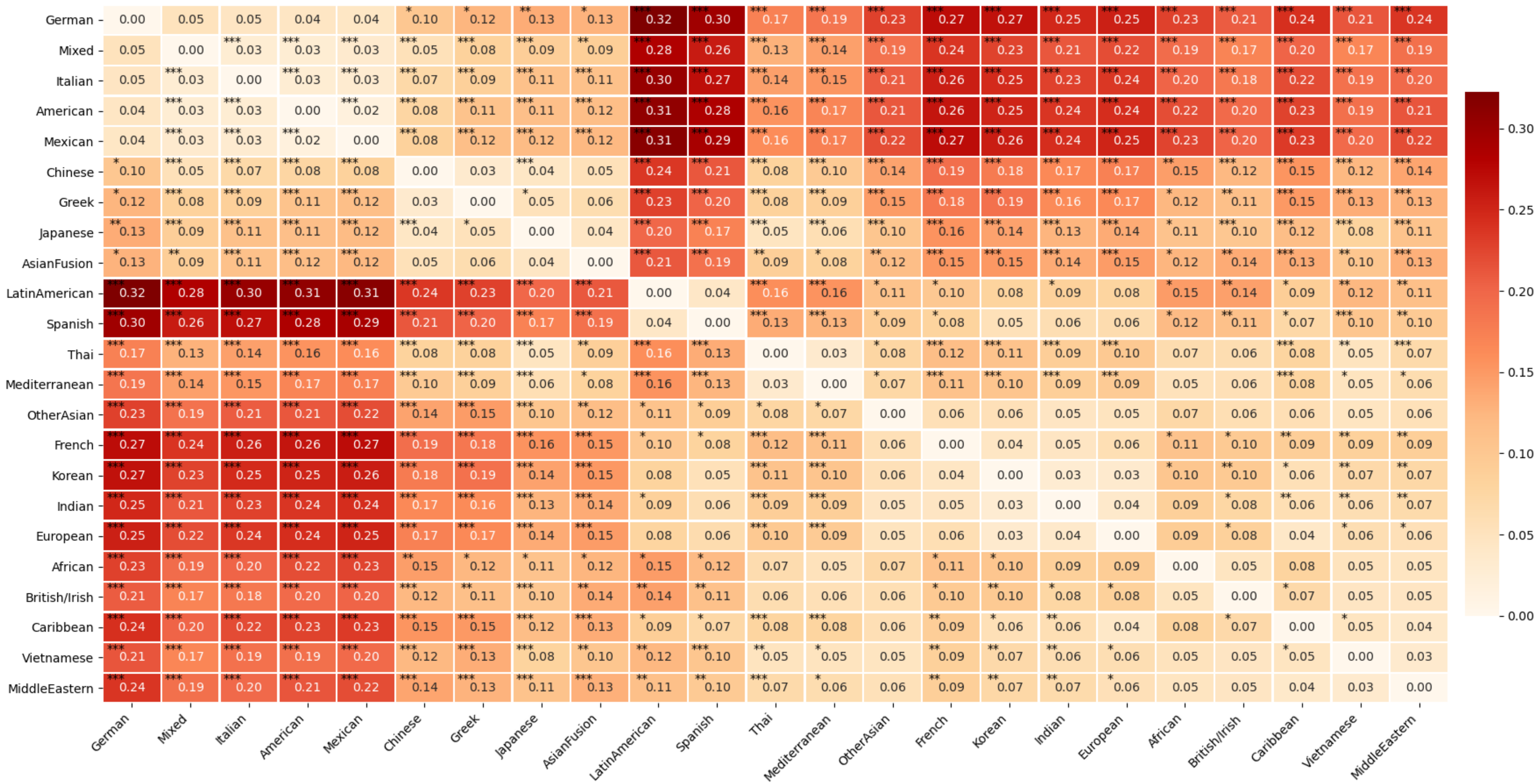

Figure S3. KS-distance statistics based on the revenue loss of restaurants across 23 restaurant types

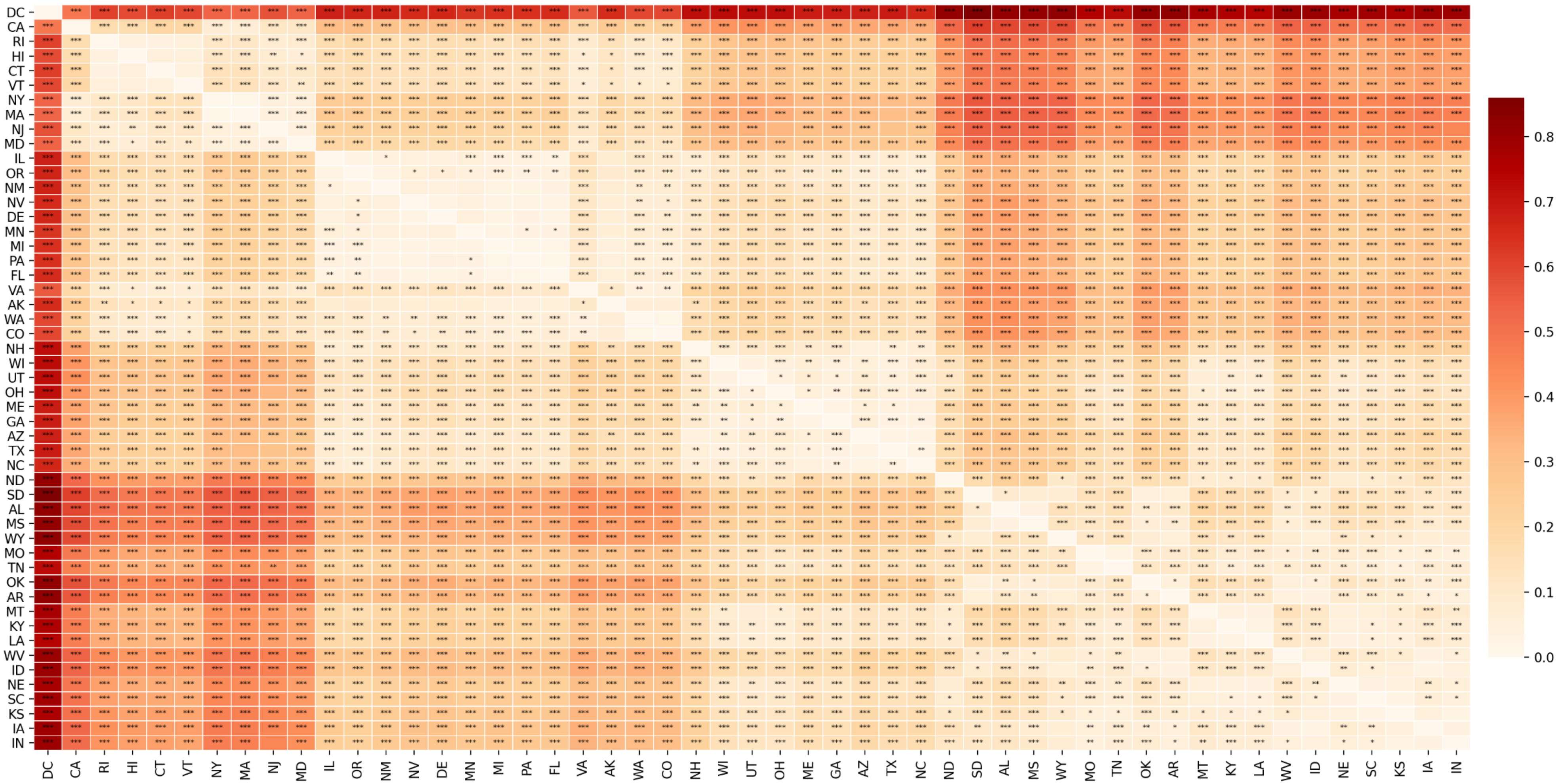

Figure S4. KS-distance statistics based on the revenue loss of restaurants across 50 states

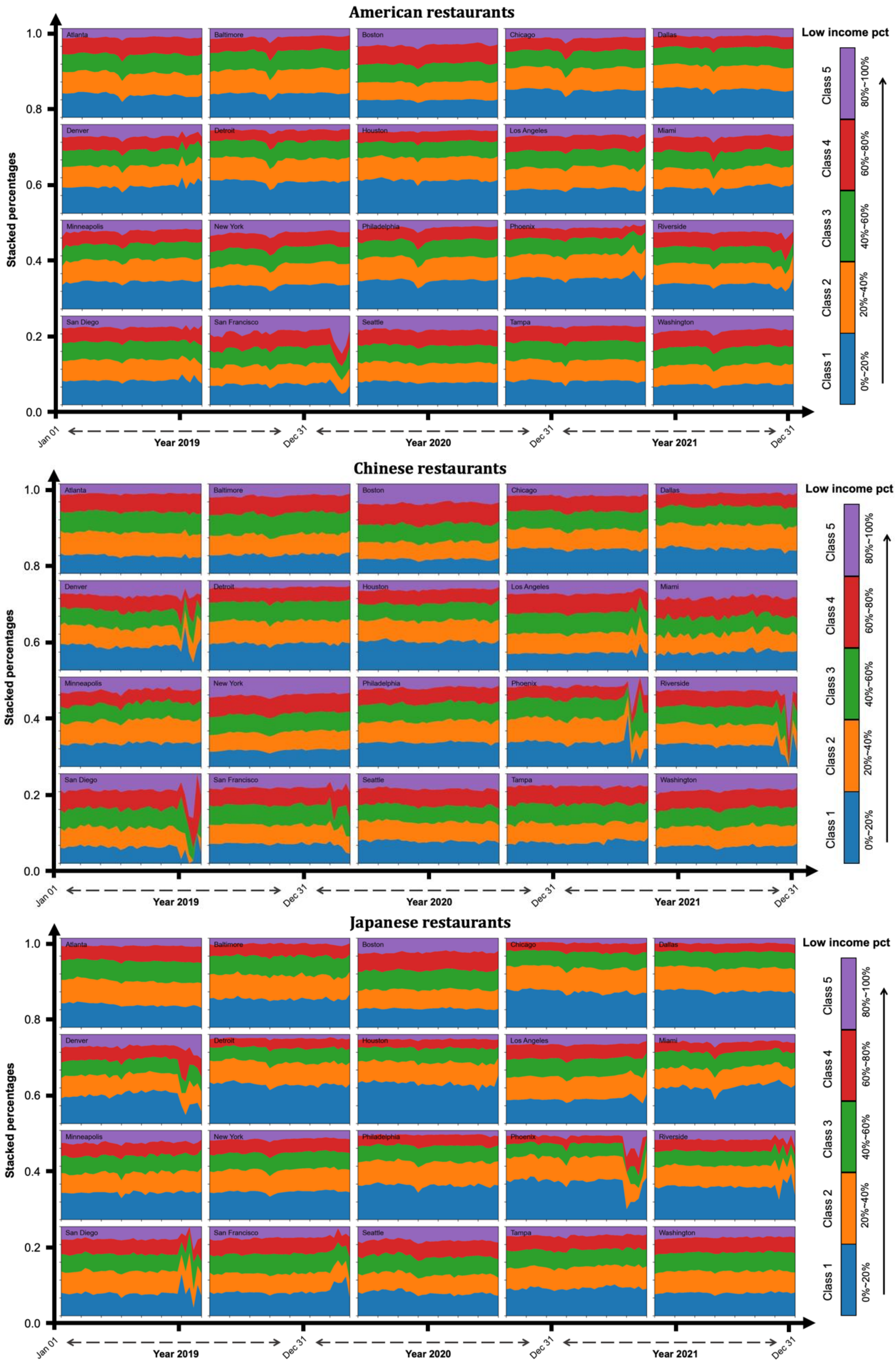

Figure S5. Statistical distribution of customers' origins (at the level of census block groups) divided by five classes based on the proportion of low-income populations for American, Chinese and Japanese restaurants. Note: The enlarged X, Y axis (bold ones) apply to each of the small graph.

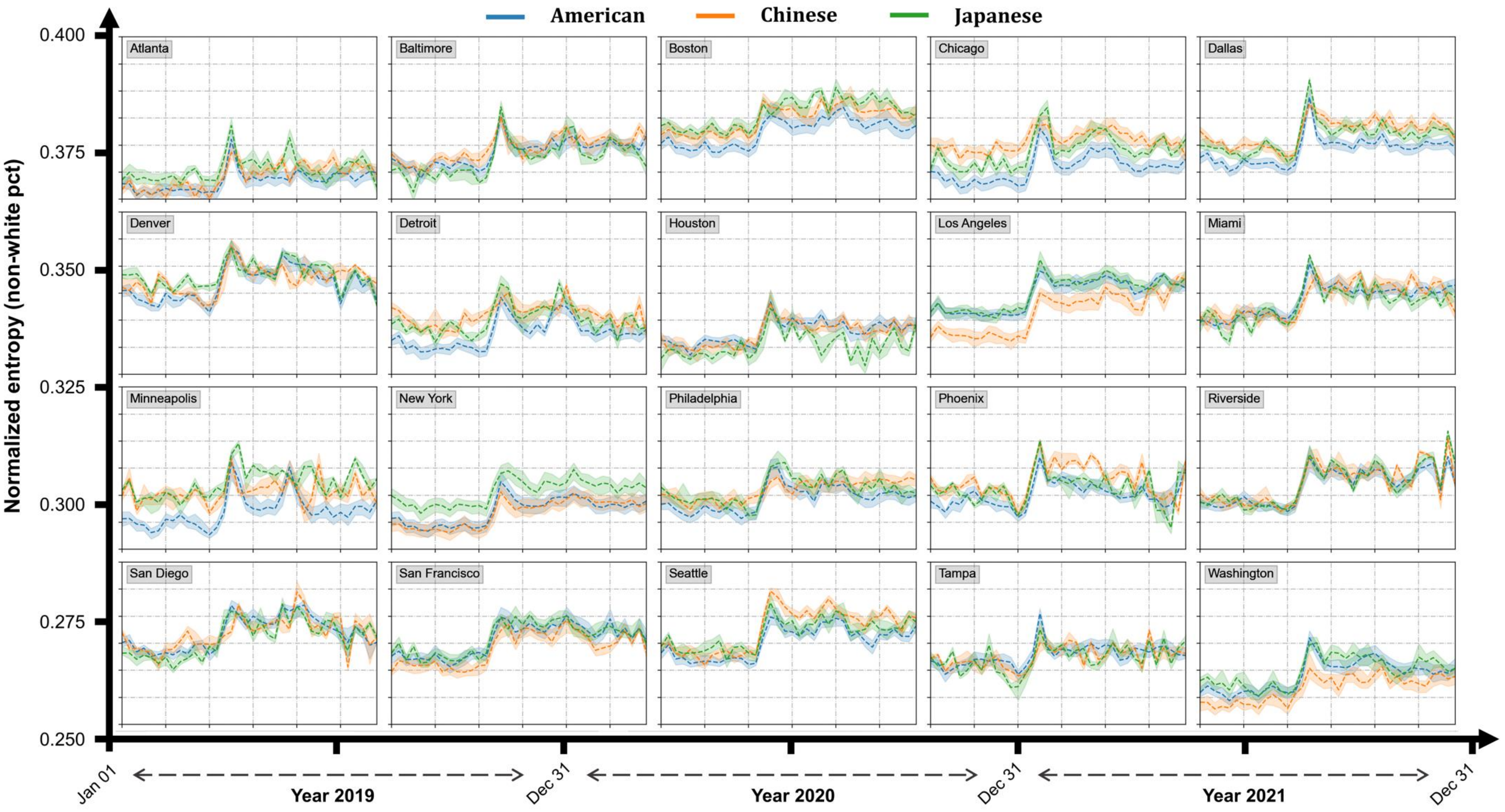

Figure S6. Entropy index used to detect the change of the areal characteristics (the proportion of non-white populations) of customers' origins after the COVID-19 outbreak. Note: The enlarged X, Y axis (bold ones) apply to each of the small graph.

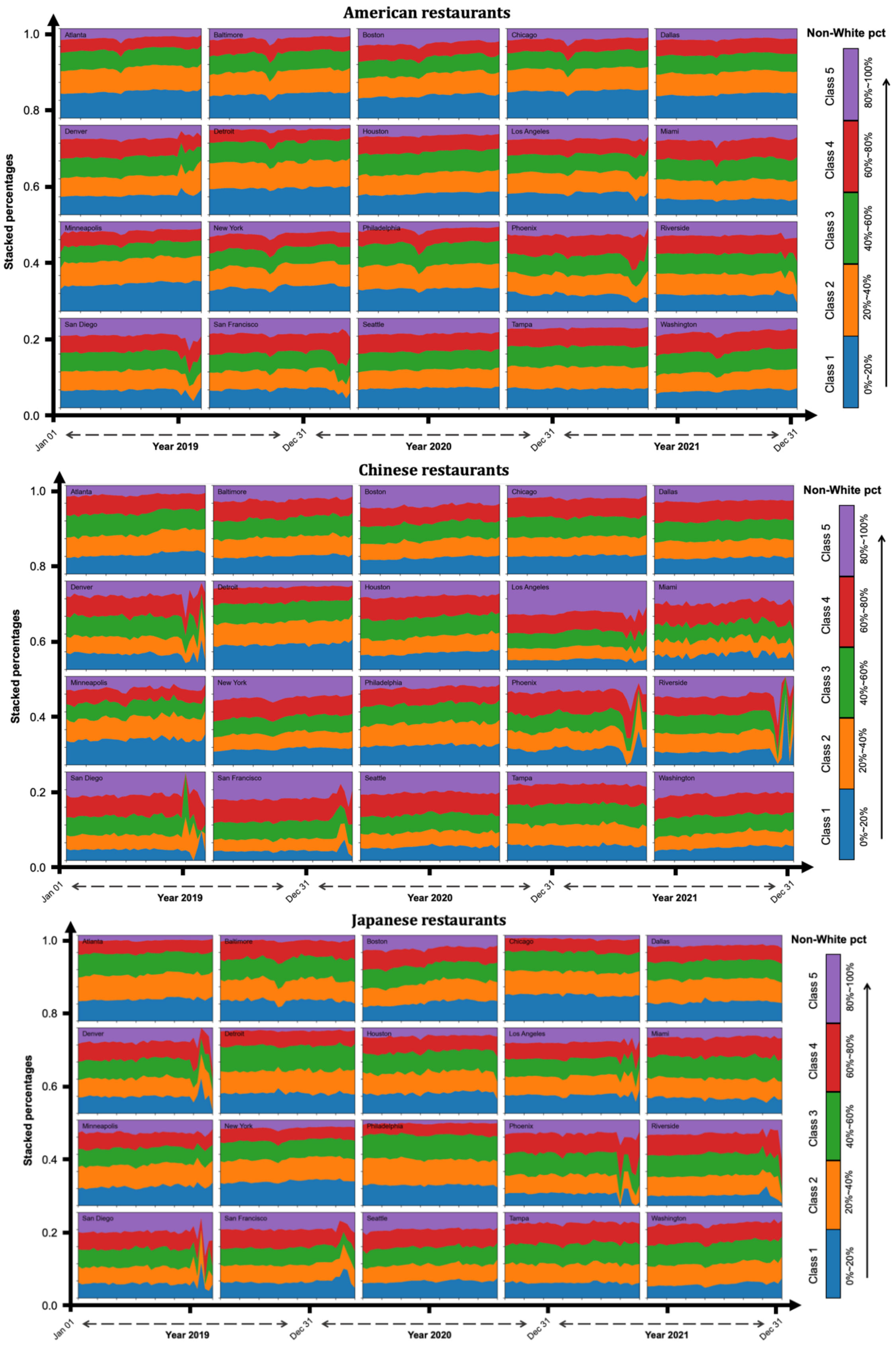

Figure S7. Statistical distribution of customers' origins (at the level of census block groups) divided by five classes based on the proportion of non-White populations for American, Chinese and Japanese restaurants. Note: The enlarged X, Y axis (bold ones) apply to each of the small graph.

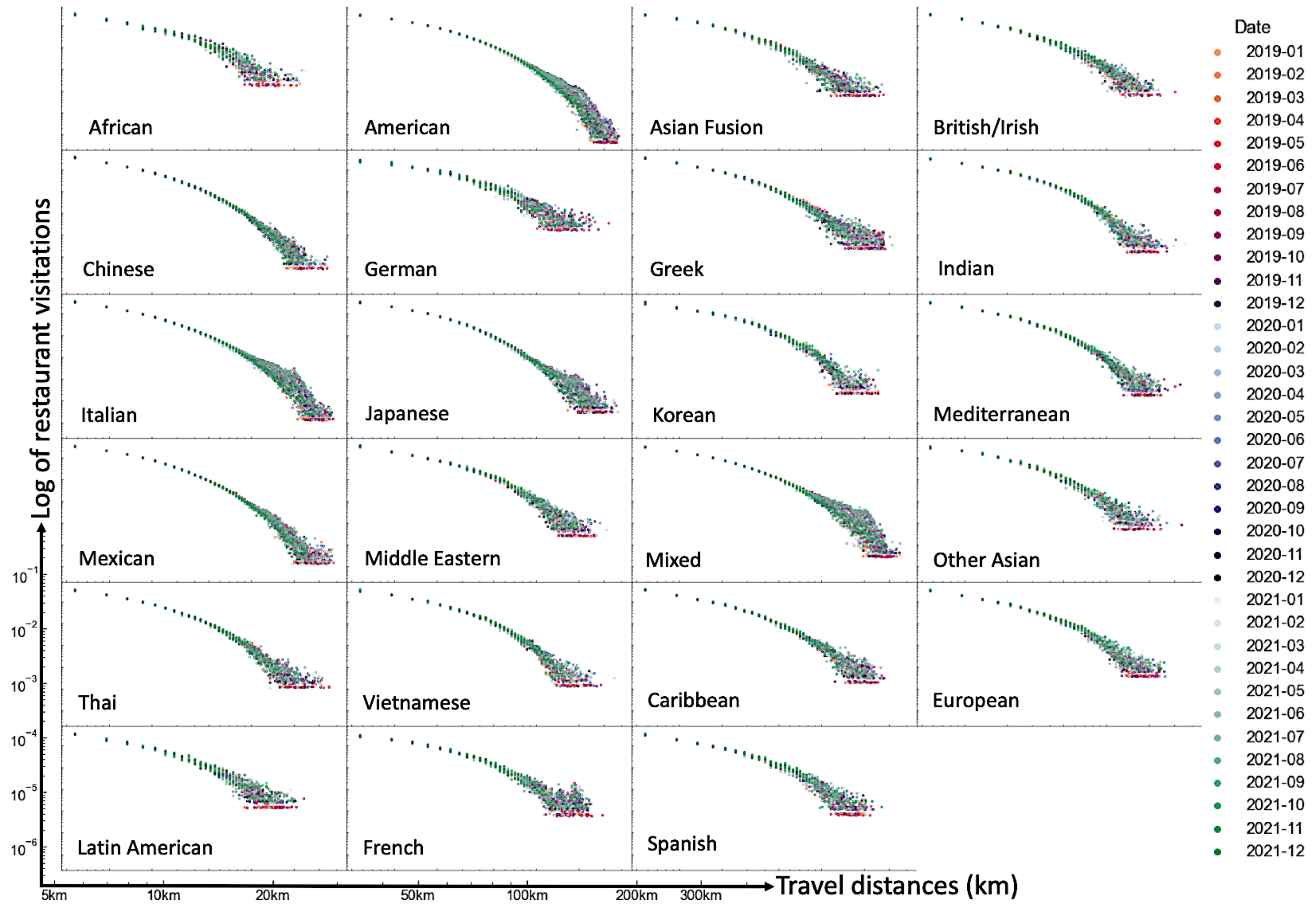

Figure S8. Relationship between travel distances (X) and the log of restaurant visitations by restaurant type (Y) for total 23 restaurant types. Note: The enlarged X, Y axis (bold ones) apply to each of the small graph.

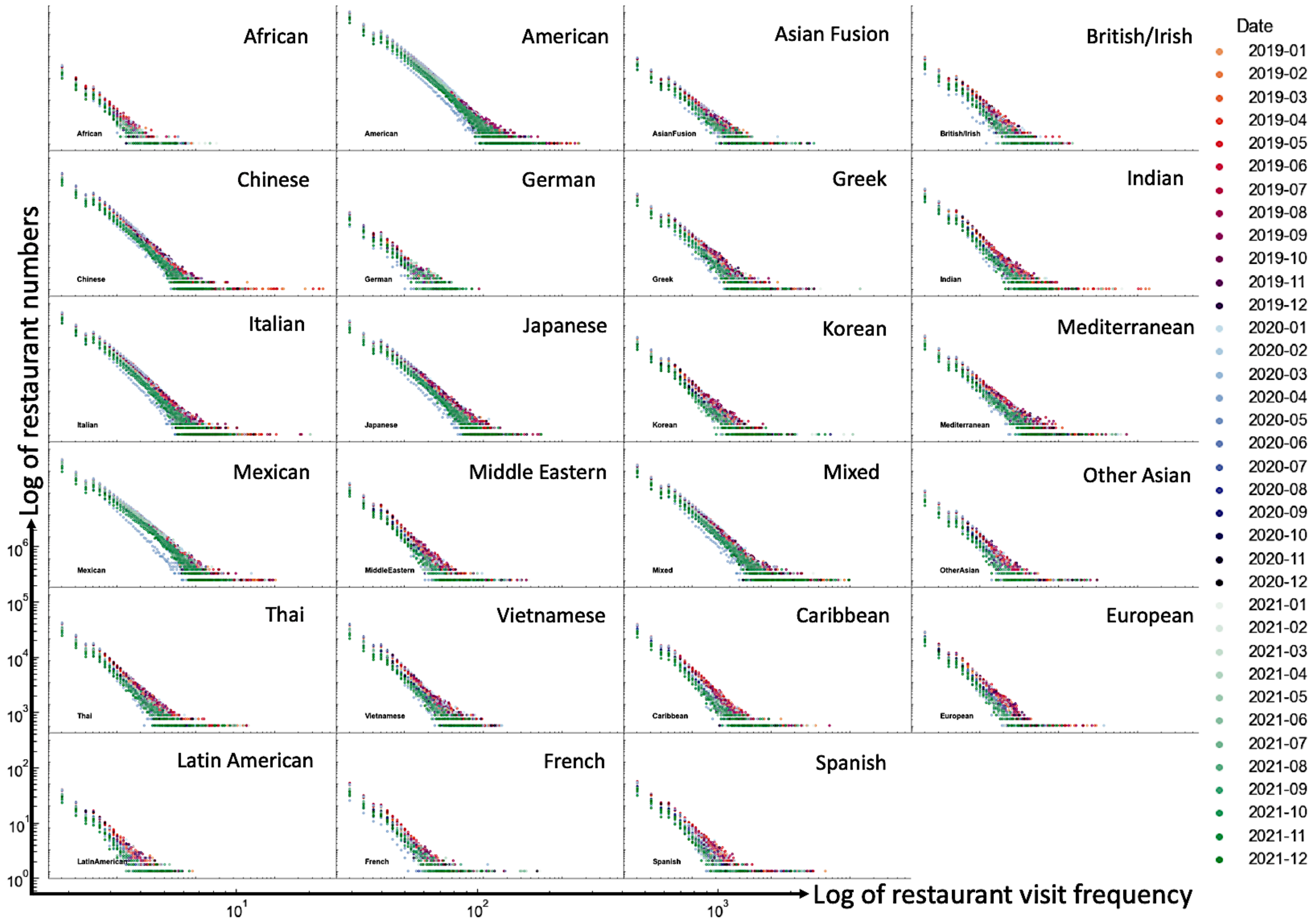

Figure S9. Relationship between the log of restaurant visit frequency (X) and the log of restaurant numbers (Y) for total 23 restaurant types. Note: The enlarged X, Y axis (bold ones) apply to each of the small graph.

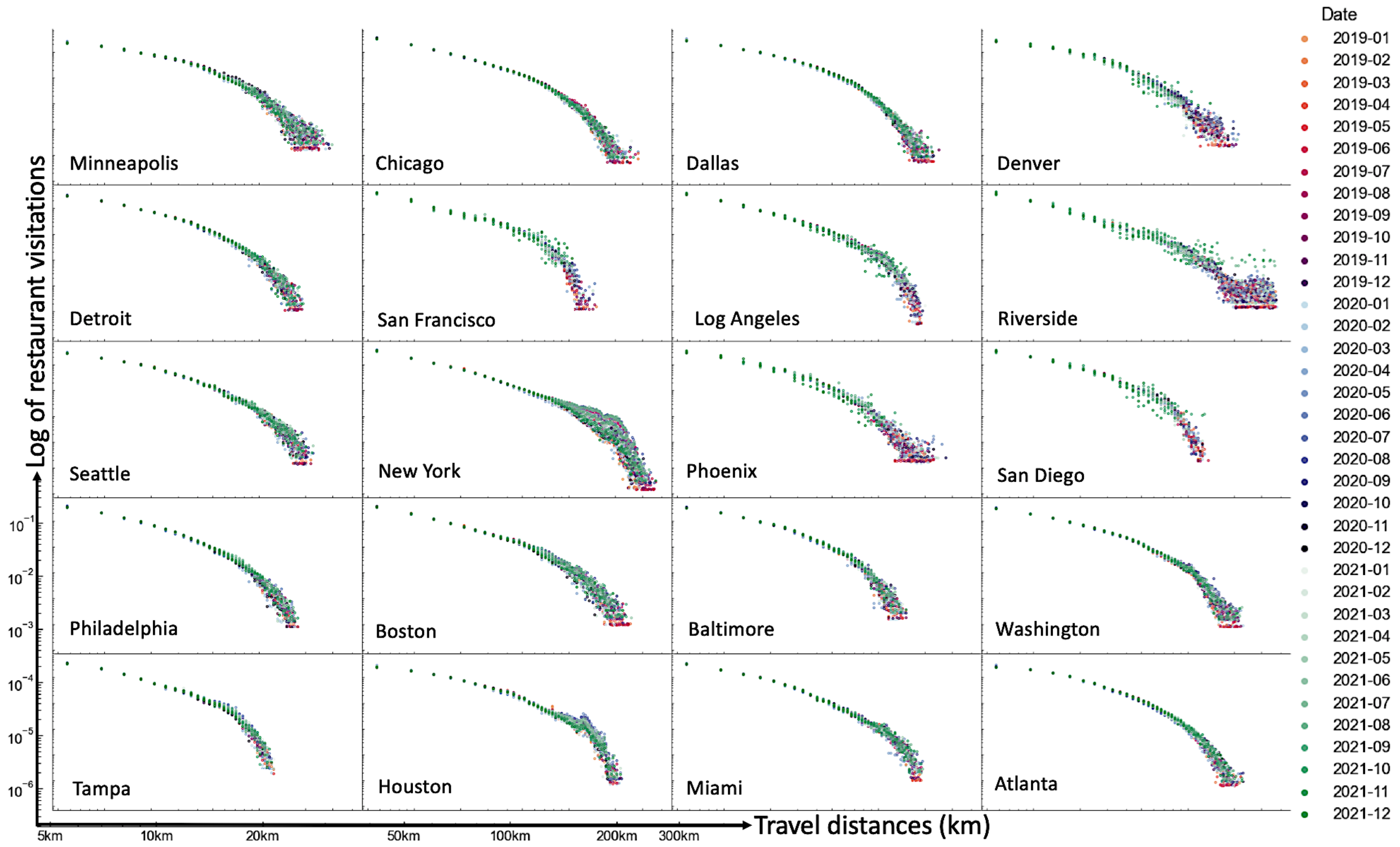

Figure S10. Relationship between travel distances (X) and the log of restaurant visitations (Y) for total 20 MSAs. Note: The enlarged X, Y axis (bold ones) apply to each of the small graph.

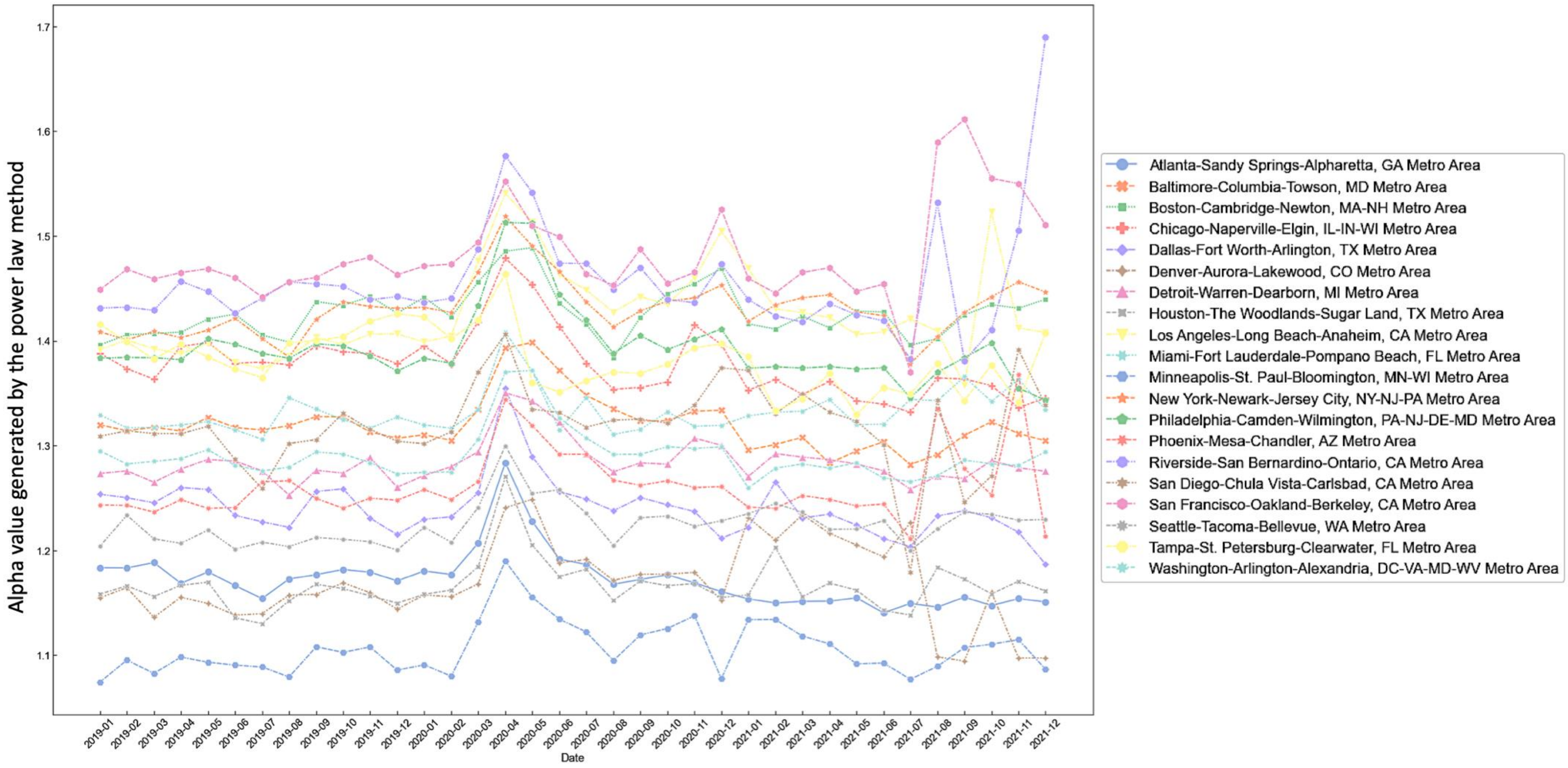

Figure S11. Alpha values generated by the power law method based on travel distances and the log of restaurant visitations by month for total 20 MSAs

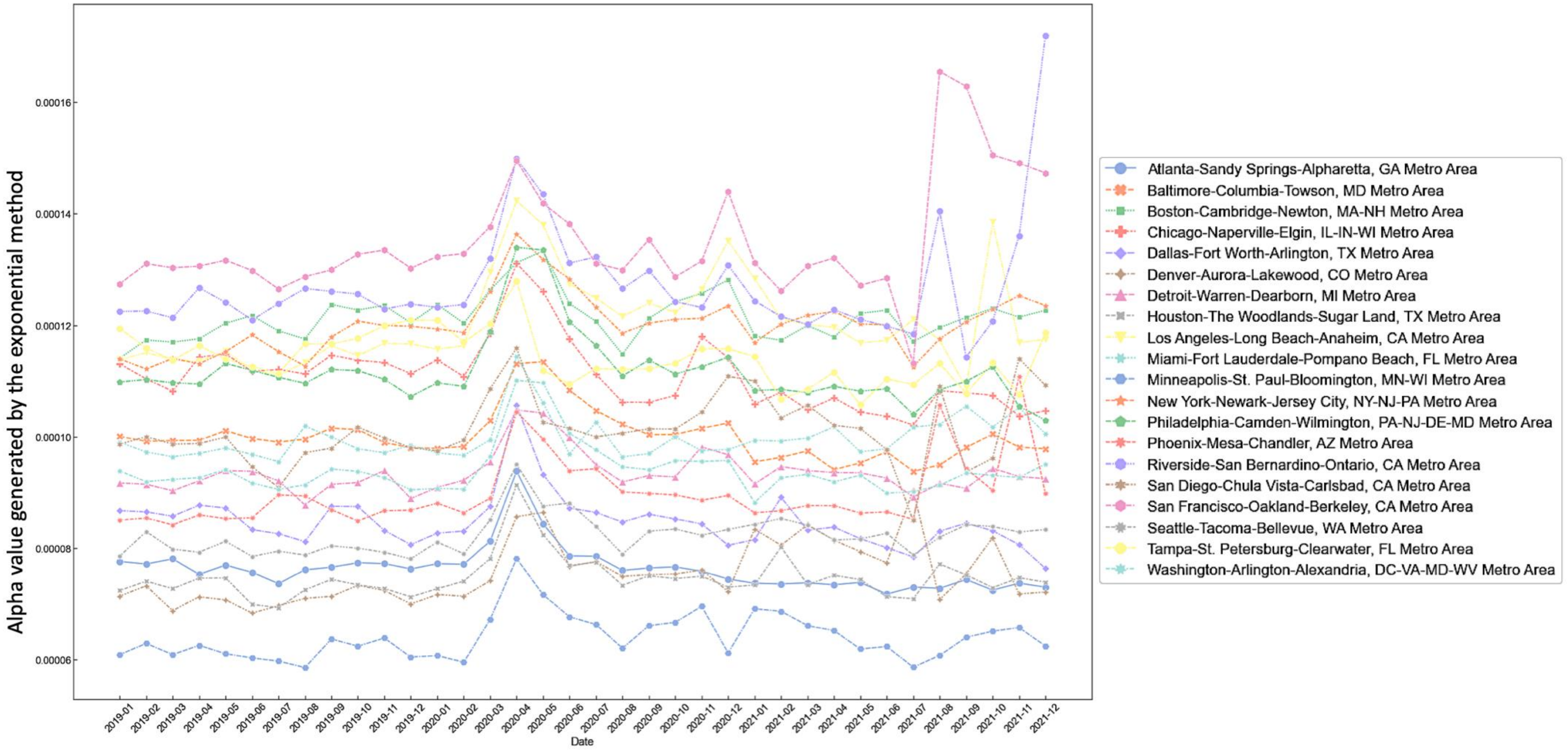

Figure S12. Alpha values generated by the exponential method based on travel distances and the log of restaurant visitations by month for total 20 MSAs

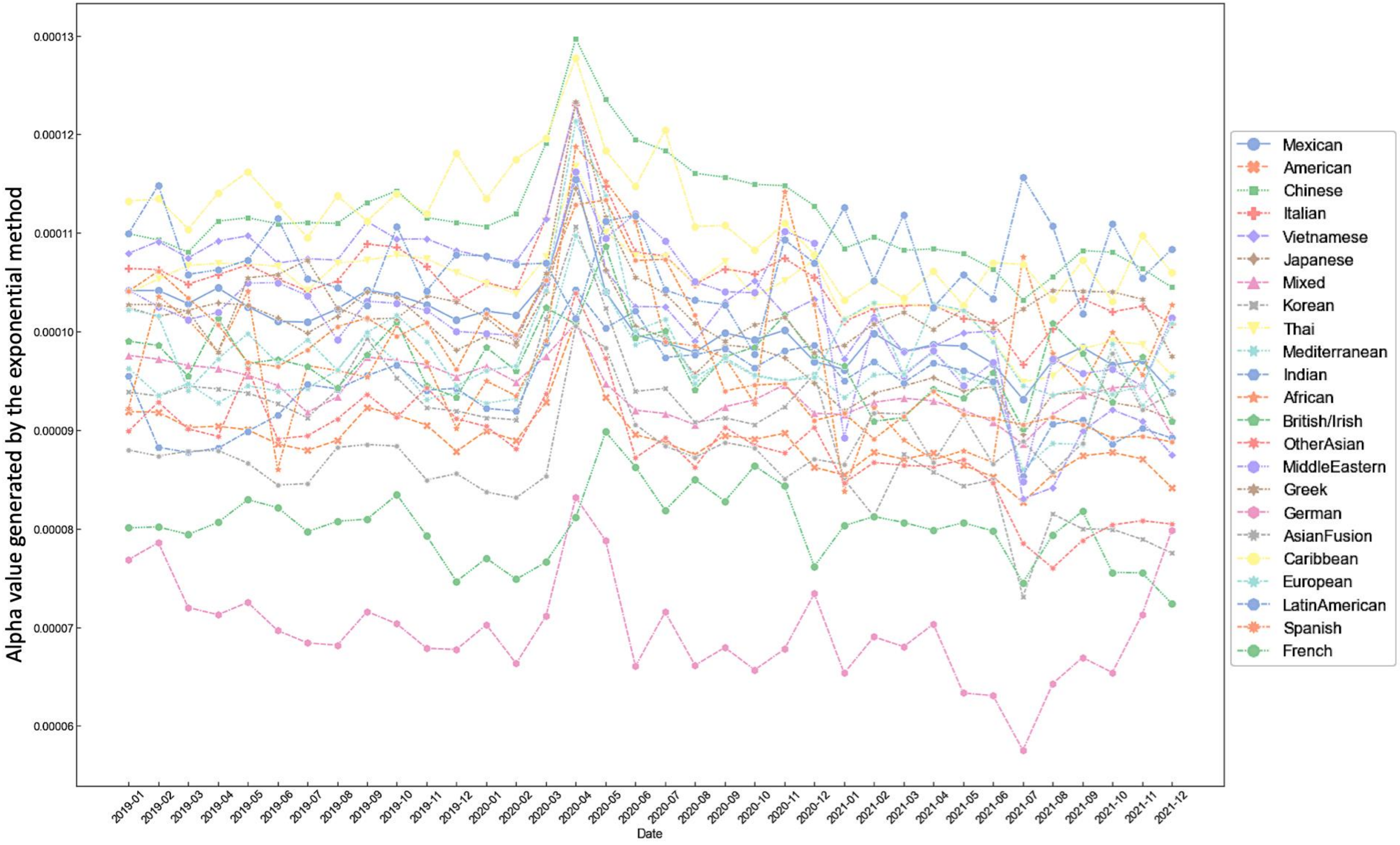

Figure S13. Alpha values generated by the power law method based on travel distances and the log of restaurant visitations by month for total 23 restaurant types

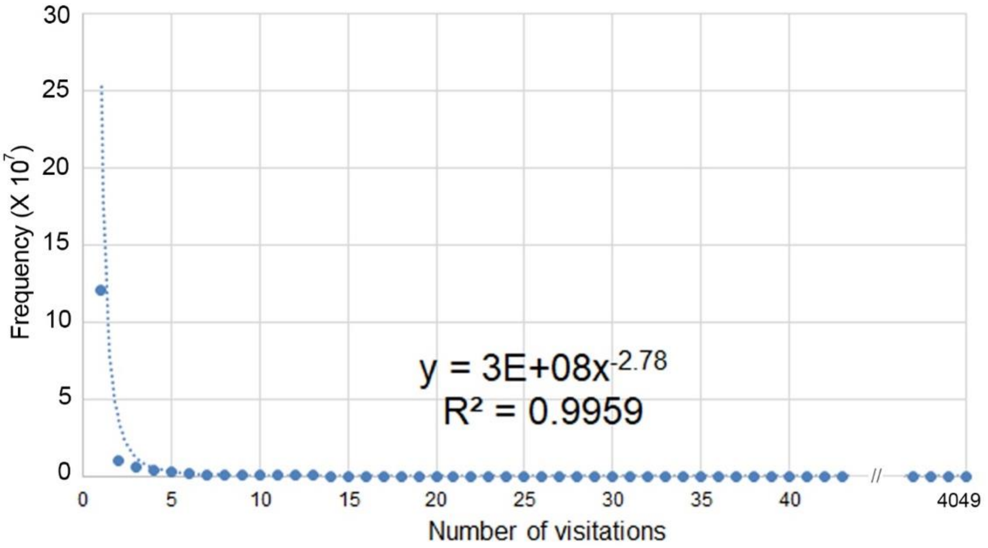

Figure S14. Curve estimation with a power form

Table S1. Estimation of the average cost by restaurant type and by state

Cost level	Frequency (%)	Estimated monetary cost ($)
$ (<=10)	64091 (30.6)	10
$$ (11–30)	89239 (42.6)	20
$$$ (31–60)	5205 (2.5)	45
$$$$(> 61)	689 (0.3)	60
Not Given	50388 (24)	To be estimated by the weighted average cost by state and by restaurant type (Eq S1)

Table S2. Reclassification of restaurant types

Type	Number	Percentage	Combination of restaurant types classified originally by Yelp
American	84204	40.17	Bagels, barbeque, burgers, chicken wings, fried chicken shops, cupcakes, fast food, fish & chips, hot dogs, pancakes, popcorn, sandwiches, southern, steakhouses.
Italian	32935	15.71	Gelato, Pasta, Pizza, Sicilian
Mexican	26027	12.42	Mexican
Mixed	15846	7.56	Bed & Breakfast, Bakeries, Bars, Brunch, Buffets, Cafes, Caterers, Cocktail Bars, Coffee & Tea, Comfort Food, Delis, Desserts, Diners, Lounges, Meat Shops, Seafood, Wine & Spirits
Chinese	15393	7.34	Cantonese, Dim Sum, Hainan, Hong Kong Style Café, Hot Pot, Kombucha, Shanghainese, Singaporean, Szechuan, Taiwanese
Japanese	10601	5.06	Japanese
Thai	4143	1.98	Thai
Indian	2764	1.32	Indian
Vietnamese	2662	1.27	Vietnamese
Caribbean	2509	1.20	Cuban, Dominican, Puerto Rican, Haitian

Mediterranean	2269	1.08	Mediterranean
Middle Eastern	1686	0.80	Afghan, Arabian, Egyptian, Falafel, Kebab, Pakistani, Persian/Iranian, Syrian, Turkish, Uzbek, Halal, Kosher
Korean	1630	0.78	Korean
European	1346	0.64	Armenian, Austrian, Belgian, Czech, Ethiopian, Fondue, Hungarian, Polish, Portuguese, Russian, Salvadoran, Scandinavian, Swiss, Ukrainian
Greek	1321	0.63	Greek
French	785	0.37	French
Other Asian	780	0.37	The minority of Asian cuisines including Bangladeshi, Burmese, Cambodian, Filipino, Himalayan, Indonesian, Laotian, Malaysian, Mongolian, Nepalese, Sri Lankan
Asian Fusion	669	0.32	The mixture of major Asian cuisines including Chinese, Japanese, Korean, and Pan Asian
Spanish	654	0.31	Spanish
British/Irish	497	0.24	British, Irish, Scottish, Ethiopian, Fondue, Hungarian, Polish, Portuguese, Russian, Salvadoran, Scandinavian, Swiss, Ukrainian
Latin American	357	0.17	Argentine, Brazilian, Colombian, Venezuelan, Peruvian
German	278	0.13	German
African	256	0.12	Lebanese, Moroccan, Nicaraguan, Senegalese, Somali, South African.
Total	209612	100	

Table S3. Restaurant visitations and frequency before and after the curve estimation

Visitation	Frequency	
	**Before estimation**	**After estimation**
2	To be estimated	87,040,460 (72.33%)
3	To be estimated	23,809,892 (19.79%)
4	120,341,708 (Break down to visitations across two, three and four)	9,491,356 (7.89%)
5	10,056,224	Remain the same
6	5,809,417	
7	3,571,173	
8	2,341,474	
9	1,622,074	
10	1,177,019	
11	884,664	
12	682,060	
13	539,286	
14	433,823	
15	351,958	
16	288,932	
17	241,392	
18	202,643	

… …		
4049	1	

Table S4. Statistical summary of restaurant visitation records retrieved from SafeGraph

Month	Visitations	Month	Visitations	Month	Visitations
2019-01	17,892,499	2020-01	23,649,969	2021-01	12,072,806
2019-02	17,686,345	2020-02	21,957,875	2021-02	11,936,664
2019-03	21,711,833	2020-03	14,310,861	2021-03	16,135,002
2019-04	21,559,962	2020-04	6,079,014	2021-04	16,328,471
2019-05	23,845,153	2020-05	10,552,373	2021-05	17,923,337
2019-06	23,663,757	2020-06	14,788,242	2021-06	17,547,819
2019-07	22,404,890	2020-07	15,559,109	2021-07	12,026,678
2019-08	23,540,640	2020-08	16,220,466	2021-08	10,791,691
2019-09	22,065,092	2020-09	15,107,920	2021-09	13,066,967
2019-10	21,026,178	2020-10	15,925,237	2021-10	10,764,119
2019-11	20,311,490	2020-11	12,753,212	2021-11	12,837,198
2019-12	22,210,949	2020-12	13,056,933	2021-12	7,675,183
Total	596,985,934				
